# Ureaplasma parvum impaired semen quality improves after doxycycline treatment in selected patients: a cohort study

**DOI:** 10.1186/s12610-025-00284-z

**Published:** 2025-09-04

**Authors:** Harm-Henning Lindhof, Bernhard Homey

**Affiliations:** Department of Dermatology, Medical Faculty, University Hospital Düsseldorf, Heinrich-Heine-University Düsseldorf, Moorenstrasse 5, 40225 Duesseldorf, Germany

**Keywords:** Infection, Semen, Fertility, *Ureaplasma*, Doxycycline, Infection, Sperme, Fertilité, Ureaplasma, Doxycycline

## Abstract

**Background:**

Infections of the urogenital tract are recognized as potential contributors to male subfertility or infertility. *Ureaplasma parvum* is frequently detected in semen samples, yet its specific impact on semen quality and the potential benefit of antibiotic treatment remain uncertain.

**Results:**

In a retrospective cohort of 3,464 semen samples collected for fertility evaluation, *Ureaplasma parvum* was identified in 12.4% of cases using a multiplex PCR assay. Semen volume was significantly lower in infected individuals compared to uninfected controls. However, no significant differences were observed in sperm concentration, motility, morphology, or inflammatory markers. Among 124 men with documented pathogen eradication and follow-up semen analysis, doxycycline treatment led to a significant increase in sperm concentration (4.01 ± 4.69 to 9.20 ± 15.63 million/ml, *p* = 0.01) and motility (18.97 ± 16.04% to 29.66 ± 22.18%, *p* = 0.002). Peroxidase-positive leukocytes declined (1.84 ± 0.74 to 0.68 ± 0.79 million/ml, *p* = 0.005). In contrast, no significant changes were observed in men with normal baseline semen parameters.

**Conclusion:**

The mere detection of *Ureaplasma parvum* does not appear to compromise semen quality and may not warrant routine antibiotic treatment. Nevertheless, in *Ureaplasma parvum*-positive patients with abnormal semen parameters and elevated inflammatory markers, targeted antibiotic therapy may improve sperm quality. These findings support a selective treatment strategy, emphasizing clinical context and inflammatory status rather than routine screening or treatment of all infected individuals.

## Résumé

**Contexte:**

Les infections de l’appareil urogénital sont reconnues comme des contributeurs potentiels à l’hypofertilité ou à l’infertilité masculine. *Ureaplasma parvum* est fréquemment détecté dans les échantillons de sperme, mais son impact spécifique sur la qualité du sperme et le bénéfice potentiel du traitement antibiotique restent incertains.

**Résultats:**

Dans une cohorte rétrospective de 3 464 échantillons de sperme recueillis pour évaluation de la fertilité, *Ureaplasma parvum* a été identifié dans 12,4% des cas à l’aide d’un test PCR multiplex. Le volume de sperme était significativement plus faible chez les hommes infectés que chez les témoins non infectés. Cependant, aucune différence significative n’a été observée pour la concentration, la motilité, la morphologie ou les marqueurs inflammatoires des spermatozoïdes. Chez 124 hommes pour lesquels l’éradication d’agents pathogènes et l’analyse de sperme de suivi ont été documentées, le traitement par doxycycline a entraîné une augmentation significative de la concentration de spermatozoïdes (de 4,0 ± 4,7 à 9,2 ± 15,6 millions/ml, *p* = 0,01) et de la motilité ( de 19,0 ± 16,0% à 29,7 ± 22,2%, *p* = 0,002). Les leucocytes positifs à la peroxydase ont diminué (de 1,84 ± 0,74 à 0,68 ± 0,79 million/ml, *p *= 0,005). En revanche, aucun changement significatif n’a été observé chez les hommes dont les paramètres de sperme de base étaient normaux.

**Conclusion:**

La simple détection d’*Ureaplasma parvum* ne semble pas compromettre la qualité du sperme et peut ne pas justifier un traitement antibiotique de routine. Néanmoins, chez les patients positifs à *Ureaplasma parvum* présentant des paramètres de sperme anormaux et des marqueurs inflammatoires élevés, une antibiothérapie ciblée peut améliorer la qualité du sperme. Ces résultats soutiennent une stratégie de traitement sélective, mettant l’accent sur le contexte clinique et l’état inflammatoire plutôt que sur le dépistage ou le traitement de routine de toutes les hommes infectées.

## Introduction

The number of couples seeking medical assistance for infertility has steadily increased in recent years [1]. While both partners typically undergo thorough diagnostic evaluations, there is growing recognition of the importance of male factor infertility, which is implicated in approximately 50% of cases [2]. Among the various etiologies, infections of the male urogenital tract are increasingly considered to contribute to subfertility or infertility [3]. Such infections are known to adversely affect semen quality, leading to reduced sperm count, impaired motility, and a lower proportion of morphologically normal sperm cells [4].

According to the guideline developed by the German, Swiss and Austrian Society of Gynecology and Obstetrics microbiological testing is recommended when clinical signs of infection or more than one million peroxidase-positive cells per milliliter are detected in the ejaculate [5]. In our andrology unit the diagnostic protocol includes comprehensive microbiological screening for *Neisseria gonorrhoeae*, *Chlamydia trachomatis*, *Ureaplasma urealyticum*, *Ureaplasma parvum*, *Mycoplasma hominis*, and *Mycoplasma genitalium* via multiplex polymerase chain reaction (PCR), in addition to the standard semen analysis based on World Health Organization (WHO) guidelines. A positive microbiological result typically leads to antibiotic treatment aimed at improving semen parameters.

*Ureaplasma parvum* is frequently detected in semen samples, yet it is generally considered a commensal organism in the urogenital tract [6]. Nevertheless, the clinical significance of *Ureaplasma parvum* in men with unexplained infertility remains unclear. Some studies suggest that asymptomatic infections may contribute to subclinical inflammation and reduced semen quality, while others report no measurable effect [7, 8].

The aim of this study was to investigate whether *Ureaplasma parvum* infections are associated with impaired semen quality and whether targeted treatment with doxycycline can lead to measurable improvements. We paid special attention to *Ureaplasma parvum*-positive patients with abnormal baseline semen parameters and elevated inflammatory markers, as this subgroup may be particularly susceptible to infection-related fertility impairment.

## Material and methods

### Study population

This retrospective cohort study was conducted at the Andrology Department of the University Hospital Düsseldorf. We included men who presented for fertility evaluation between January 2016 and December 2019. All participants underwent a standard andrological assessment, including at least one semen analysis according to World Health Organization (WHO) guidelines and microbiological analysis for the detection of *Neisseria gonorrhoeae*, *Chlamydia trachomatis*, *Ureaplasma urealyticum*, *Ureaplasma parvum*, *Mycoplasma hominis*, and *Mycoplasma genitalium* using a validated in-house multiplex PCR assay [9]. In addition to PCR testing, patients with more than 1 million peroxidase-positive leukocytes per milliliter underwent conventional culture-based diagnostics to screen for potential infection with Gram-positive and Gram-negative bacteria. This step was performed to identify relevant pathogens beyond the detection range of the PCR panel. Protozoal and viral pathogens were not assessed and represent a limitation of this study.

Patients with known causes of infertility (e.g., chromosomal abnormalities or history of gonadotoxic chemotherapy), those with high-grade varicoceles requiring surgery, or those who did not adhere to the recommended period of 2–7 days of ejaculatory abstinence were excluded. No patient had received antibiotics within 3 months prior to sample collection. All included patients were asymptomatic at the time of evaluation.

All patients with Ureaplasma parvum infection received doxycycline at a dose of 200 mg once daily for 14 days. They were advised to inform their sexual partners of the positive test result, and partner testing with simultaneous treatment—if indicated—was strongly recommended to minimize the risk of reinfection and to support sustained microbiological eradication. Patients were counseled to abstain from unprotected sexual activity during the treatment period and until microbiological cure had been confirmed in both themselves and their partners. Treatment success was assessed by repeat PCR testing two weeks after completion of antibiotic therapy. Semen samples were collected again approximately three months after treatment.

### Semen analysis

Semen samples were collected by masturbation into sterile, non-toxic containers after 2–7 days of sexual abstinence. All samples were immediately incubated at 37 °C and processed within 60 min. Semen analysis was performed according to the WHO 5th edition guidelines [10] and included.

- Volume (measured by graduated tube)
- pH (determined by indicator strips)
- Sperm concentration (counted in a Neubauer chamber)
- Motility (assessed by phase-contrast microscopy at 400 × magnification), and
- Morphology (evaluated using Papanicolaou staining and strict WHO criteria).

Peroxidase-positive leukocytes were measured using the ortho-toluidine method as described in the WHO laboratory manual [10]. This test identifies granulocytes based on their peroxidase activity and expresses results in million per milliliter (Mio/ml). Values ≥ 1 Mio/ml were considered elevated according to the WHO criteria [10].

In addition, leukocytes per 100 spermatozoa were recorded as part of routine laboratory documentation. However, this parameter is not included in WHO recommendations and was analyzed descriptively only.

### Statistical analysis

Data analysis was performed using Python libraries (NumPy, Pandas, and SciPy), with continuous variables presented as mean ± standard deviation. The Mann–Whitney U test was applied for comparisons between groups when data were non-normally distributed, while normally distributed data were assessed using the Student’s t-test. Paired pre- and post-treatment semen parameters were compared using paired t-tests. A significance level of p < 0.05 was set for all analyses. Given the exploratory nature of this study, p-values are presented descriptively. A post-hoc power analysis was conducted to evaluate the study's ability to detect significant changes in semen parameters following treatment.

Analyses of non-standard parameters such as leukocytes per 100 spermatozoa were performed on an exploratory basis and are interpreted with caution due to the lack of formal WHO validation.

## Results

### Cohort characteristics

Out of 3,464 semen samples collected between 2016 and 2019, 2,427 were primary analyses, and 1,037 were follow-up samples. Among the 2,427 initial samples, 371 (15.3%) tested positive for at least one urogenital pathogen by multiplex PCR. After excluding 71 cases with co-infections, 300 samples (12.4%) were positive for *Ureaplasma parvum* only and formed the study group.

The control group consisted of 2,056 age-matched semen samples with negative PCR results. There were no significant differences between the two groups regarding age (36.96 vs. 37.95 years), ejaculatory abstinence period (5.13 vs. 5.44 days), or the interval between first and second semen samples (109 vs. 106 days).

Among the 300 *Ureaplasma parvum*-positive patients, 157 had follow-up semen analyses after doxycycline treatment. In 124 of these, eradication was confirmed by negative PCR two weeks after therapy, and paired semen data before and after treatment were available.

### Baseline comparison between infected and uninfected patients

As shown in Table 1, men with *Ureaplasma parvum* had a significantly lower semen volume compared to uninfected controls (3.10 ± 1.41 vs. 3.45 ± 1.79 ml, p < 0.001). No significant differences were found in sperm concentration (*p* = 0.90), motility (*p* = 0.39), morphology (*p* = 0.64), peroxidase-positive leukocytes (*p* = 0.21), or leukocytes per 100 spermatozoa (*p* = 0.48).

**Table 1:** Comparison of the primary semen analysis of patients with Ureaplasma parvum vs uninfected control group. All data presented as mean ± standard deviation. Statistical comparisons between groups were performed using the two-sided unpaired t-test

	Control group (*n* = 2056)	***Ureaplasma parvum*** (*n* = 300)	*p*-Value
Volume (ml)	3.45 ± 1.79	3.10 ± 1.41	< 0.001
Concentration of spermatozoa (Mio/ml)	48.6 ± 61.6	49.0 ± 52.27	0.90
Motility (%)	43.03 ± 15.33	43.82 ± 14.81	0.39
Normally formed sperm (%)	1.25 ± 1.60	1.30 ± 1.65	0.64
Peroxidase-positive cells (Mio/ml)	0.18 ± 0.58	0.22 ± 0.50	0.21
Leukocytes (per 100 Spermatozoa)	2.24 ± 7.62	2.53 ± 6.51	0.48

### Treatment effect in patients with abnormal baseline parameters

**Fig. 1:**
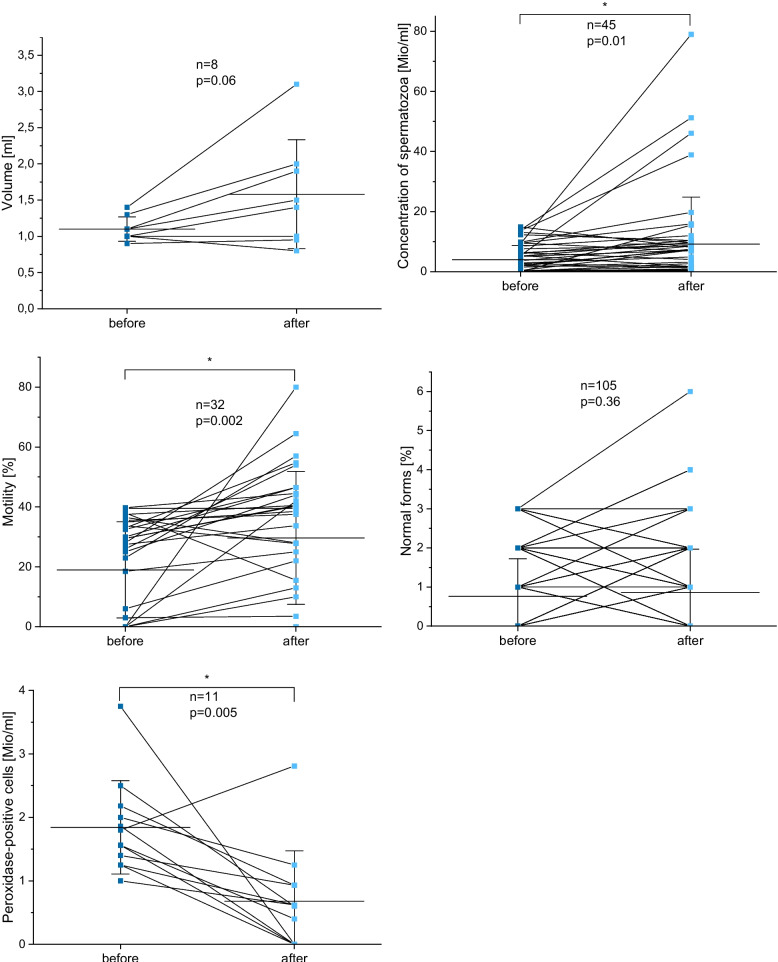
Paired pre- and post-treatment semen parameters in Ureaplasma parvum-positive patients with abnormal baseline values who achieved microbiological cure following doxycycline therapy. Only patients with values below or above the WHO reference thresholds at baseline were included for each respective parameter. Each panel displays individual patient trajectories for semen volume, sperm concentration, progressive motility, normal morphology, and peroxidase-positive leukocytes. Paired data points are connected by lines. Horizontal lines indicate median values; error bars represent the standard error of the mean. Statistical comparisons were performed using paired t-tests

Among the 124 patients with confirmed pathogen eradication, not all exhibited abnormal baseline values across all semen parameters. Therefore, Fig. 1 presents paired pre- and post-treatment data exclusively *Ureaplasma parvum*-positive individuals with pathological baseline findings and elevated peroxidase-positive leucocytes. The number of patients included in each analysis is indicated accordingly.

Significant improvements were observed in sperm concentration, which increased from 4.01 ± 4.69 to 9.20 ± 15.63 million/ml (*n* = 45; *p* = 0.01), and in progressive motility, which rose from 18.97 ± 16.04% to 29.66 ± 22.18% (*n* = 32; *p* = 0.002). A marked decrease in peroxidase-positive leukocytes was also noted, from 1.84 ± 0.74 to 0.68 ± 0.79 million/ml (*n* = 11; *p* = 0.005).

In contrast, no statistically significant changes were observed in semen volume among patients with an initial volume below 1.5 ml (*n *= 8; *p* = 0.06), nor in sperm morphology in those with less than 4% normal forms at baseline (*n* = 105; *p* = 0.36).

### Exploratory observation: azoospermia

Among ten patients who were azoospermic at baseline, three had detectable spermatozoa in the follow-up semen sample. Due to the absence of repeated baseline testing and the small sample size, these findings are reported descriptively only and should be interpreted with caution.

### Post-hoc power analysis

A post-hoc power analysis confirmed the robustness of these findings. With a sample size of 124 patients and a significance level of p < 0.05, the study had 95.6% power to detect a moderate effect size (Cohen's d = 0.33) in sperm concentration. In the case of motility, the power was 99.96%, corresponding to a moderate to large effect size (Cohen's d = 0.48). The study had a power of 100% to detect a very large effect size (Cohen's d = 1.47) in peroxidase-positive cells, and 100% power with a moderate to large effect size (Cohen's d = 0.64) in semen volume. However, for morphology, the effect size was small (Cohen's d = 0.08) and the study had a power of only 14.6%, indicating that the sample size was insufficient to detect changes in morphology with confidence.

## Discussion

In this retrospective cohort study, we evaluated the association between *Ureaplasma parvum* infection and semen quality, and investigated the effects of doxycycline treatment in men with confirmed eradication. While *Ureaplasma parvum* was the most frequently detected pathogen in our cohort, its overall impact on semen parameters was limited. Semen volume was reduced in infected men, but no significant differences were found in sperm concentration, motility, morphology, or inflammatory markers when compared to uninfected controls.

Importantly, targeted antibiotic treatment led to measurable improvement in *Ureaplasma parvum*-positive patients with initially abnormal semen parameters. Sperm concentration, motility, and peroxidase-positive leukocytes significantly improved after doxycycline therapy in this subgroup. No changes were observed in men with normal baseline parameters, supporting a selective rather than universal treatment approach.

### Comparison with existing literature

Most existing studies have investigated the role of *Ureaplasma spp.* in male infertility without distinguishing between U. parvum and U. urealyticum [11]. More recent literature has focused predominantly on U. urealyticum, showing significant associations with impaired semen parameters and elevated inflammatory markers. In contrast, data specific to U. parvum remain limited [12, 13].

Recent studies have demonstrated a significant impact of *Ureaplasma urealyticum* on semen quality and inflammatory makers, but no impact on the outcome of intrauterine insemination or in vitro fertilization could be shown [14, 15]. In contrast, a meta-analysis reported no statistically significant association between *Ureaplasma parvum* colonization and male infertility [16].

While most studies have focused on inflammatory or oxidative mechanisms, recent evidence suggest that *Ureaplasma parvum* may also exert its effects through epigenetic modulation. Galdiero et al. demonstrated that normospermic, *Ureaplasma parvum*-positive men exhibited significantly altered microRNA expression, associated with decreased motility and increased oxidative stress. Target gene analysis suggested that these miRNAs regulate lipid kinase activity, indicating a potential role in membrane remodeling and motility regulation [17].

### Immunological perspective and pathophysiology

Infections of the male genital tract elicit an inflammatory response, primarily driven by the release of pro-inflammatory cytokines like TNF-α, IL-1β, and IL-6 from activated macrophages [18–21]. These cytokines stimulate the generation of reactive oxygen species (ROS), which in turn damage spermatozoa by disrupting membrane integrity, inducing DNA fragmentation, and impairing mitochondrial function. Elevated ROS levels are a known cause of oxidative stress, a major contributor to impaired sperm motility and morphology [22].

Beyond ROS production, cytokines play a broader role in testicular dysfunction. IL-1 and IL-6 have been shown to disrupt the seminiferous epithelium by altering Sertoli cell tight junctions, thereby compromising the blood-testis barrier. This can lead to germ cell detachment and impaired spermatogenesis. Moreover, cytokines negatively influence Leydig cell function, resulting in decreased testosterone synthesis [19].

Chronic inflammation, often associated with IL-4, promotes fibrotic remodeling of testicular tissue TGF-β signaling. This process involves the activation of fibroblast and extracellular matrix deposition, which can reduce testicular elasticity and impair the microvascular environment essential for spermatogenesis. The cumulative effect of these pathways is a deterioration in semen quality and hormonal dysregulation [18].

Doxycycline, the antibiotic used in this study, is known not only for its antimicrobial activity but also for its anti-inflammatory properties. It inhibits matrix metalloproteinases, reduces leukocyte migration, and modulates cytokine expression, including suppression of TNF-α and IL-6. Additionally, doxycycline possesses direct ROS-scavenging effects, thereby attenuating oxidative damage in the testicular environment [23]. These mechanisms may help explain the observed improvements in sperm quality following treatment, especially in men with elevated inflammatory markers.

The observed therapeutic response suggests that subclinical inflammation may be a critical mediator of infertility in *Ureaplasma parvum*-positive men. Therefore, markers such as peroxidase-positive leukocytes may serve not only as diagnostic indicators but also as tools for stratifying treatment decisions.

### Limitations

Several limitations must be acknowledged. First, the retrospective design may introduce selection bias, and semen analysis was not standardized to multiple baseline samples in all patients, as recommended by WHO. Second, while we excluded patients with co-infections within the PCR panel, other potential uropathogens such as anaerobes, protozoa, and viruses were not systematically tested, and may have confounded a minority of cases. Third, leukocytes per 100 spermatozoa were reported as an exploratory parameter only, since no validated cut-off exists for clinical relevance. Fourth, although doxycycline therapy was associated with improvements in certain azoospermic patients, the small sample size and lack of multiple baseline samples preclude definitive conclusions.

Finally, spontaneous recovery cannot be excluded in some patients, particularly in cases with fluctuating or borderline semen parameters.

Inclusion of a post-hoc power analysis supports the robustness of our findings, indicating that the observed improvements in sperm concentration, motility, and inflammatory markers were unlikely due to chance alone. However, as such analyses are inherently constrained by the observed effect sizes and sample variability, the results should be interpreted with caution. Future prospective studies with predefined power calculations are needed to validate these findings in targeted patient subgroups.

## Conclusions

While *Ureaplasma parvum* is frequently detected in semen samples, its presence alone is not associated with significant impairment of sperm concentration, motility, or morphology in the general infertile male population. Routine screening and antibiotic treatment are therefore not warranted in asymptomatic men with normal semen profiles.

However, in selected Ureaplasma positive-patients with abnormal semen parameters and elevated inflammatory markers, *Ureaplasma parvum* eradication lead to clinically meaningful improvements in sperm quality and declined peroxidase-positive leukocytes. These findings highlight the importance of integrating microbiological testing with inflammatory diagnostics, such as peroxidase-positive leukocyte quantification, in the andrological workup.

Future prospective studies should further investigate the role of local cytokine profiles and oxidative stress parameters in identifying men who may benefit from targeted antimicrobial therapy.

## Data Availability

The datasets used and/or analyzed during the current study are available from the corresponding author on reasonable request.

## Abbreviations

PCR: Polymerase chain reaction
ROS: Reactive oxygen species
WHO: World Health Organization
